# Fast activation maximization for molecular sequence design

**DOI:** 10.1186/s12859-021-04437-5

**Published:** 2021-10-20

**Authors:** Johannes Linder, Georg Seelig

**Affiliations:** 1 Paul G. Allen School of Computer Science and Engineering, University of Washington, Seattle, USA; 2 Department of Electrical and Computer Engineering, University of Washington, Seattle, USA

**Keywords:** Activation maximization, Sequence design, DNA, RNA, Protein, Deep learning, Design, Gradient ascent, Neural network, Optimization

## Abstract

**Background:**

Optimization of DNA and protein sequences based on Machine Learning models is becoming a powerful tool for molecular design. Activation maximization offers a simple design strategy for differentiable models: one-hot coded sequences are first approximated by a continuous representation, which is then iteratively optimized with respect to the predictor oracle by gradient ascent. While elegant, the current version of the method suffers from vanishing gradients and may cause predictor pathologies leading to poor convergence.

**Results:**

Here, we introduce Fast SeqProp, an improved activation maximization method that combines straight-through approximation with normalization across the parameters of the input sequence distribution. Fast SeqProp overcomes bottlenecks in earlier methods arising from input parameters becoming skewed during optimization. Compared to prior methods, Fast SeqProp results in up to 100-fold faster convergence while also finding improved fitness optima for many applications. We demonstrate Fast SeqProp’s capabilities by designing DNA and protein sequences for six deep learning predictors, including a protein structure predictor.

**Conclusions:**

Fast SeqProp offers a reliable and efficient method for general-purpose sequence optimization through a differentiable fitness predictor. As demonstrated on a variety of deep learning models, the method is widely applicable, and can incorporate various regularization techniques to maintain confidence in the sequence designs. As a design tool, Fast SeqProp may aid in the development of novel molecules, drug therapies and vaccines.

**Supplementary Information:**

The online version contains supplementary material available at 10.1186/s12859-021-04437-5.

## Background

Rational design of DNA, RNA and protein sequences has enabled the rapid development of a wide range of biomolecules, including functional or stably folded proteins [1–3], optimized promoter sequences [4], active enzymes [5] and *de novo* antibody components [6, 7]. These design principles are now starting to be applied to specific therapeutic domains, for example AAV gene therapy [8], antimicrobial peptides [9] and vaccines [10, 11]. A number of Machine Learning methods have been explored for sequence design, such as Genetic Algorithms [12], Simulated Annealing [3, 13], Bayesian optimization [14], Particle swarms [15–17] and population-based methods [18]. Most design methods are guided by predictive models, often based on deep learning, that reliably relate sequence to fitness or function [19–36]. More recently, methods combining adaptive sampling or other conditioning techniques with deep generative networks have been used to model distributions of sequences with desired properties [6, 9, 37–42], including Deep Exploration Networks (DENs) which were developed by our group. While powerful, these methods first require selecting an appropriate generative network and tuning several hyper-parameters.

Perhaps the simplest and most direct approach to sequence design based on a differentiable fitness predictor is to optimize the input pattern by gradient ascent [29, 37, 43–46]. This approach is commonly known as activation maximization and uses the gradient of the neural network output to make incremental changes to the input. However, gradient ascent cannot be directly applied to discrete sequence data. Several extensions have been proposed to rectify this. Killoran et al. [44] used a softmax layer to turn the sequences into continuous relaxations. In previous work, we developed *SeqProp* which uses straight-through (ST) gradients to optimize discrete samples [29]. However, as our results indicate below, both methods converge slowly. Furthermore, continuous input relaxations may cause predictor pathologies leading to poor designs.

Here, we develop *Fast SeqProp*, a gradient-based design method that combines discrete nucleotide sampling and straight-through approximation with normalization across the parameters of the sampling distributions. We hypothesized that these modifications would overcome the issue of slow convergence encountered by previous methods. To test this idea, we systematically compared Fast SeqProp to prior methods on a range of DNA and protein design tasks, including the design of strong enhancers, 5’UTRs, alternative polyadenylation signals and protein structures. We also examined whether methods based on direct optimization (such as activation maximization) in general reach higher fitness scores than conditioning of generative models when there is a low degree of epistemic uncertainty. Finally, we explored techniques for regularizing activation maximization such that the designed sequences do not drift too far from the original training data distribution.

Fast SeqProp demonstrated up to a 100-fold optimization speedup, and improved optima, on the design tasks compared to prior methods based on activation maximization. We validated designs by scoring them with models that were not used during optimization. We also found that our method can outperform global search heuristics such as Simulated Annealing as well as more recent methods based on generative models. Unlike the latter approaches, Fast SeqProp does not require training of an independent generator. It is thus model–free, making it easy to use when designing smaller sequence sets. Moreover, Fast SeqProp can incorporate many different regularization techniques to maintain confidence in its designs, such as regularization based on a variational autoencoder (VAE) and optimization of probabilistic predictor models that are capable of estimating their uncertainty.

### Activation maximization for biological sequences

Given a sequence-predictive neural network $\mathcal {P}$ and an initial input pattern $\varvec{x}^{(0)}$, the gradient ascent method seeks to maximize the predicted fitness $\mathcal {P}(\varvec{x}) \in \mathbb {R}$ by tuning the input pattern $\varvec{x}$:

1 $$\begin{aligned} \max _{\varvec{x}} \mathcal {P}(\varvec{x}) \end{aligned}$$

Assuming $\mathcal {P}$ is differentiable, we can compute the gradient $\nabla _{\varvec{x}} \mathcal {P}(\varvec{x})$ with respect to the input and optimize $\varvec{x}$ by updating the variable with a small step $\eta \in \mathbb {R}$ in the direction of the fitness gradient [47]:

2 $$\begin{aligned} \varvec{x}^{(t+1)} \leftarrow \varvec{x}^{(t)} + \eta \cdot \nabla _{\varvec{x}} \mathcal {P}(\varvec{x}) \end{aligned}$$

However, sequences are usually represented as one-hot coded patterns ($\varvec{x} \in \{0, 1\}^{N \times M}$, where *N* is the sequence length and *M* the number of channels or monomer possibilities; $M=4$ for nucleic acids and $M=20$ for proteins), and discrete variables cannot be optimized by gradient ascent. Several different reparameterizations of $\varvec{x}$ have been proposed to bypass this issue. In one of the earliest implementations, Lanchantin et al. [43] represented the sequence as an unstructured, real-valued pattern ($\varvec{x} \in \mathbb {R}^{N \times M}$) but imposed an L2-penalty on $\varvec{x}$ in order to keep it from growing too large and causing predictor pathologies. After optimization, this real-valued pattern is interpreted as a sequence logo from which samples can be drawn. However, the method was introduced mainly as a visualization tool rather than a sequence design approach. Killoran et al. [44] later introduced a softmax reparameterization, turning $\varvec{x}$ into a continuous relaxation $\sigma (\varvec{l})$:

3 $$\begin{aligned} \sigma (\varvec{l})_{ij} = \frac{ e^{\varvec{l}_{ij}} }{ \sum _{k=1}^{4} e^{\varvec{l}_{ik}} } \end{aligned}$$

Here $\varvec{l}_{ij} \in \mathbb {R}$ are differentiable *nucleotide logits*. The gradient of $\sigma (\varvec{l})$ with respect to $\varvec{l}$ is defined as:

4 $$\begin{aligned} \frac{\partial \sigma (\varvec{l})_{ij}}{\partial {\varvec{l}}_{ik}} = \sigma ({\varvec{l}})_{ik} \cdot \left( {\mathbbm{1}}_{(j = k)} - \sigma ({\varvec{l}})_{ij}\right) \end{aligned}$$

Given Eqs. 3 and 4, we can maximize $\mathcal {P}(\sigma (\varvec{l}))$ with respect to the logits $\varvec{l}$ using the gradient $\nabla _{\varvec{l}} \mathcal {P}\left( \sigma (\varvec{l})\right)$. While elegant, there are two issues with this architecture. First, the gradient in Eq. 4 becomes vanishingly small for large values of $\varvec{l}_{ij}$ (when $\sigma (\varvec{l})_{ik} \approx 0$ or $\sigma (\varvec{l})_{ij} \approx 1$), halting convergence. Second, sequence-predictive neural networks have only been trained on discrete one-hot coded patterns and the predictive power of $\mathcal {P}$ may be poor on a continuous relaxation such as $\sigma (\varvec{l})$.

Following advances in gradient estimators for discretized neurons [48, 49], we developed *SeqProp*, a version of the gradient ascent method that replaces the softmax transform $\sigma$ with a discrete, stochastic sampler $\delta$:

5 $$\begin{aligned} \delta ({\varvec{l}})_{ij} = {\mathbbm {1}}_{(Z_{i} = j)} \end{aligned}$$

Here, $Z_{i} \sim \sigma (\varvec{l})_{i}$ is a randomly drawn categorical nucleotide at the *i*th position from the (softmax) probability distribution $\sigma (\varvec{l})_{i}$. The nucleotide logits $\varvec{l}_{ij}$ can be interpreted as parameters to *N* categorical distributions, from which we sample nucleotides $\{Z_{i}\}_{i=1}^{N}$ and construct a discrete, one-hot coded pattern $\delta (\varvec{l}) \in \{0, 1\}^{N \times M}$. While $\delta (\varvec{l})$ is not directly differentiable, $\varvec{l}$ can be updated based on the estimate of $\nabla _{\varvec{l}} \mathcal {P}(\delta (\varvec{l}))$ using straight-through approximation. Rather than using the original ST estimator of Bengio et al. [48], we here adopt an estimator with theoretically better properties from Chung et al. [50] where the gradient of $\delta (\varvec{l})_{ij}$ is replaced by that of the softmax $\sigma (\varvec{l})_{ij}$:

6 $$\begin{aligned} \frac{\partial \delta (\varvec{l})_{ij}}{\partial \varvec{l}_{ik}} \approx \frac{\partial \sigma (\varvec{l})_{ij}}{\partial \varvec{l}_{ik}} = \sigma (\varvec{l})_{ik} \cdot ({\mathbbm{1}}_{(j = k)} - \sigma (\varvec{l})_{ij}) \end{aligned}$$

By sending discrete samples as input to $\mathcal {P}$ we remove any pathology that could arise from using a continuous input relaxation. But, as we show below, convergence remains almost as slow as the softmax method. Switching to the original ST estimator ($\frac{\partial \delta (\varvec{l})_{ij}}{\partial \varvec{l}_{ij}} = 1$) speeds up convergence but worsens fitness optima (see Additional file 1, Figure S1G for a comparison).

## Results

### Fast stochastic sequence backpropagation

**Fig. 1:**
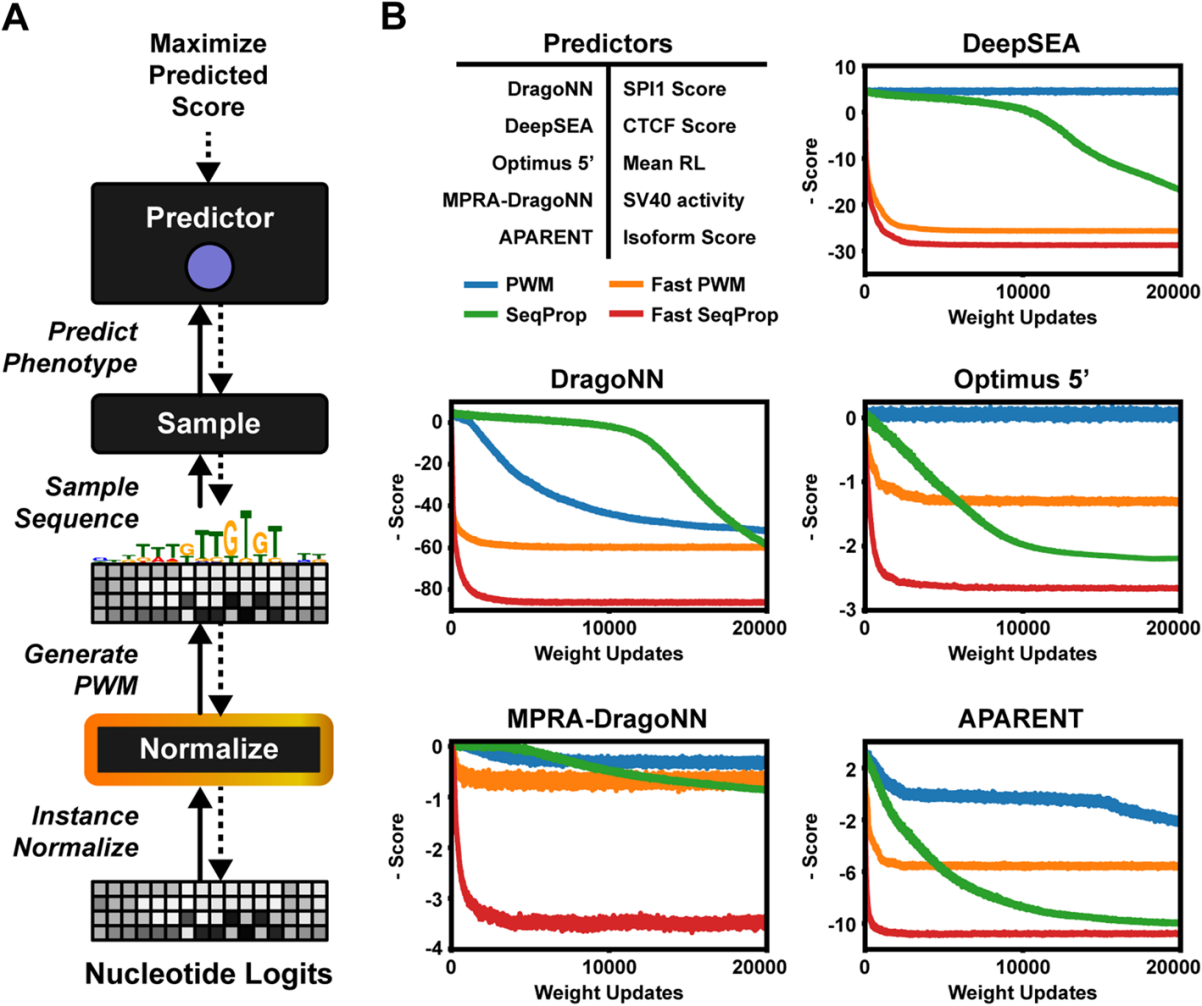
Fast activation maximization for sequence design. **a** The Fast SeqProp pipeline. A normalization layer is prepended to a softmax layer, which is used as parameters to a sampling layer. **b** Maximizing the predictors DragoNN (SPI1), DeepSEA (CTCF Dnd41), MPRA-DragoNN (SV40), Optimus 5’ and APARENT

Inspired by instance normalization in image GANs [51], we hypothesized that the main bottleneck in earlier design methods—both in terms of optimization speed and minima found—stem from overly large and disproportionally scaled nucleotide logits. Here, we mitigate this problem by normalizing the logits across positions. Specifically, we insert a normalization layer between the trainable logits $\varvec{l}_{ij}$ and the sampling layer $\delta (\varvec{l})_{ij}$ (Fig. 1a).

For DNA sequence design, where the number of one-hot channels *M* is small ($M = 4$), we use a normalization scheme commonly referred to as *instance*-normalization. In this scheme, the nucleotide logits of each channel are normalized independently across positions. Let $\bar{\mu }_{j} = \frac{1}{N} \sum _{i=1}^{N} \varvec{l}_{ij}$ and $\bar{\varepsilon }_{j} = \sqrt{\frac{1}{N} \sum _{i=1}^{N} (\varvec{l}_{ij} - \bar{\mu }_{j})^2}$ be the sample mean and deviation of logits for nucleotide *j* across all positions *i*. For each step of gradient ascent, we compute the normalized logits $\varvec{l}_{ij}^{(\text {norm})}$ as:

7 $$\begin{aligned} \varvec{l}_{ij}^{(\text {norm})} = \frac{ \varvec{l}_{ij} - \bar{\mu }_{j} }{ \bar{\varepsilon }_{j}^2 } \end{aligned}$$

Since logits with zero mean and unit variance have limited expressiveness when used as parameters to a probability distribution, we associate each channel *j* with a global scaling parameter $\gamma _{j}$ and offset $\beta _{j}$. Having an independent offset $\beta _{j}$ per channel is particularly well-suited for DNA, as nucleotides are often associated with a global preferential bias. The scaled, re-centered logits are calculated as:

8 $$\begin{aligned} \varvec{l}_{ij}^{(\text {scaled})} = \varvec{l}_{ij}^{(\text {norm})} * \gamma _{j} + \beta _{j} \end{aligned}$$

For protein sequence design, the number of one-hot channels *M* is considerably larger ($M = 20$) while the sequences often are shorter, resulting in fewer samples per channel and noisier normalization statistics. Here we found that *layer*-normalization was more stable: We compute a global mean $\bar{\mu } = \frac{1}{N \cdot M} \sum _{i=1}^{N} \sum _{j=1}^{M} \varvec{l}_{ij}$ and deviation $\bar{\varepsilon } = \sqrt{\frac{1}{NM} \sum _{i=1}^{N} \sum _{j=1}^{M} (\varvec{l}_{ij} - \bar{\mu }_{j})^2}$, and use a shared scaling factor $\gamma$ and offset $\beta$ for all *M* channels.

Given the normalized and scaled logits $\varvec{l}^{(\text {scaled})}$ as parameters for the nucleotide sampler $\delta$ defined in Eq. 5, we maximize $\mathcal {P}(\delta (\varvec{l}^{(\text {scaled})}))$ with respect to $\varvec{l}_{ij}$, $\gamma _{j}$ and $\beta _{j}$ (or $\gamma$ and $\beta$ in the context of proteins) using the softmax ST estimator from Eq. 6. The normalization removes logit drift by keeping the values proportionally scaled and centered at zero ($\mathrm {E}[l_{ij}^{(\text {norm})}] = 0$, $\mathrm {Var}[l_{ij}^{(\text {norm})}] = 1$), enabling the gradients to swap nucleotides with few updates. Furthermore, the scaling parameter $\gamma _{j}$ (or $\gamma$) adaptively adjusts the sampling entropy to control global versus local optimization: When our confidence in a particular nucleotide *j* at position *i* ($\varvec{l}_{ij}^{(\text {norm})}$) is *consistent* with its impact on fitness (shares the sign of the fitness gradient $\frac{\partial \mathcal {P}(\delta (\varvec{l}^{(\text {scaled})}))}{\partial \delta (\varvec{l}^{(\text {scaled})})_{ij}}$), the scaling parameter $\gamma _{j}$ increases, thus lowering sampling entropy. Whenever we sample inconsistent nucleotides, $\gamma _{j}$ decreases and the temperature again increases, promoting exploration. See Methods for further details.

### Maximizing nucleic acid sequence-predictive neural networks

We first evaluated our method on the task of maximizing the classification or regression scores of five DNA- or RNA-level neural networks: (1) *DragoNN*, a model trained on ChIP-seq data to predict Transcription Factor (TF) binding (in this case binding of SPI1), (2) *DeepSEA* [22], which predicts multiple TF binding probabilities and chromatin modifications (we use it here to maximize the probability of CTCF binding in the cell type Dnd41), (3) *APARENT* [29], which predicts alternative polyadenylation isoform abundance given an input polyadenylation signal, (4) MPRA-DragoNN [24], a neural network trained to predict transcriptional activity of short enhancer sequences and, finally, (5) *Optimus 5’* [25], which predicts ribosomal load (translational efficiency) of 5’ UTR sequences.

We compare our new logit-normalized, straight-through sampled sequence design method (*Fast SeqProp*) to the previous versions of the algorithm, namely the original method with continuous softmax-relaxed inputs [44] (here referred to as *PWM*) and *SeqProp*, the categorical sampling method described in [29] using a (non-normalized) gradient estimator. We also tested a logit-normalized version of the softmax-relaxed method, *Fast PWM*, in order to disentangle the individual performance contributions of the normalization scheme and the sampling scheme.

Figure 1b shows the result of using the design methods to generate maximally scored sequences for each of the five DNA-based predictors. Fast SeqProp converges to 95–99% of its minimum test loss within 2000 logit updates, and reaches 50% of the minimum loss after only 200 updates for all predictors except MPRA-DragoNN and Optimus 5’. In contrast, PWM and SeqProp do not converge within 20,000 updates. Fast SeqProp converges to up to threefold better optima than all other compared methods. In fact, Fast SeqProp reaches the same or better optima in 200 updates than the competing methods reach in 20,000 updates for DragoNN, MPRA-DragoNN and DeepSEA, marking a 100x speedup. For Optimus 5’ and APARENT, the speedup is 20x-50x. In addition to gradient-based methods, we demonstrate improved performance compared to discrete search algorithms such as Simulated Annealing (see Additional file 1, Figure S1A-B).

In the Additional file 1, we provide additional technical comparisons of Fast SeqProp to previous activation maximization methods. For example, In Figure S1C, we demonstrate that certain sequence-predictive neural networks suffer from out-of-distribution (OOD) pathologies on continuous sequence relaxations as input, explaining the poor performance of the PWM design method. We further show that adding an entropy penalty to the PWM method still cannot close the performance gap to Fast SeqProp (Figure S1D) and that the Softmax ST estimator outperforms Gumbel Sampling on a number of tasks (Figure S1E). Finally, we show that Fast SeqProp appears robust to the choice of optimizer parameters (Figure S1F) and that the Softmax ST estimator outperforms the original ST estimator (Figure S1G).

### Recapitulating *cis*-regulatory biology with activation maximization

**Fig. 2:**
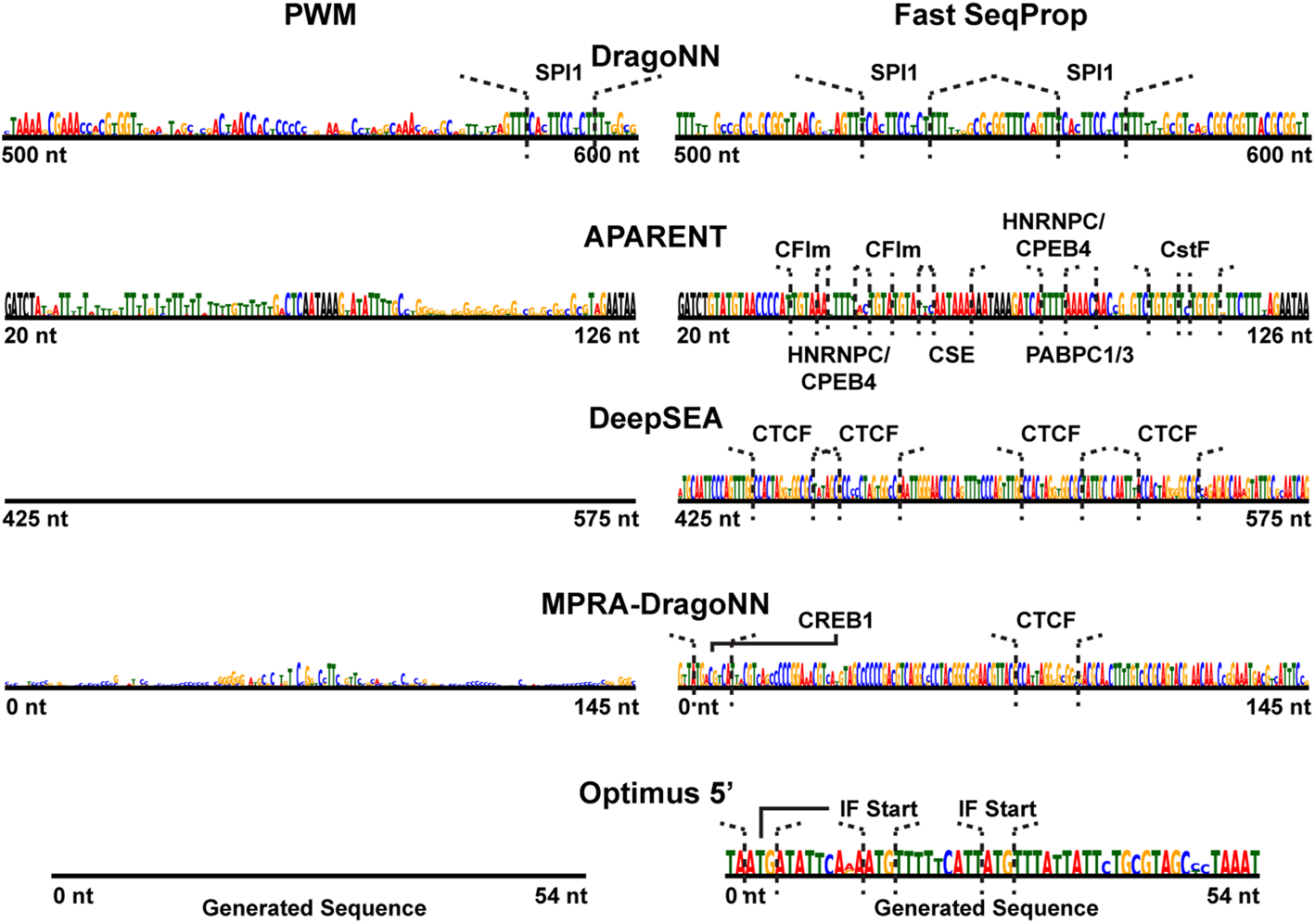
Example designed sequences. Softmax sequences (PSSMs) generated by the PWM and Fast SeqProp methods after 20,000 updates of gradient ascent updates with default optimizer parameters (Adam). The logit matrices $\varvec{l}$ were uniformly randomly initialized prior to optimization. Identified cis-regulatory motifs annotated above each sequence

In Fig. 2 we compare example sequence optimizations of the PWM and Fast SeqProp methods. As can be seen, even after 20, 000 updates, the PWM method has not converged for most of the tested predictors. In contrast, we find plenty of *cis*-regulatory motifs in the converged sequences generated by Fast SeqProp. Since our method was tasked with *maximizing* the predicted score of each model, we would expect to find enhancing motifs and regulatory logic embedded in the sequences which give rise to these extreme model responses.

For example, when maximizing DragoNN, Fast SeqProp generates multiple SPI1 binding motifs [52]. For APARENT, Fast SeqProp generates CFIm binding motifs, dual CSE hexamers, and multiple cut sites with CstF binding sites. These are all regulatory motifs known to enhance cleavage and polyadenylation by stimulating recruitment of the polyadenylation machinery [53–56]. For DeepSEA, Fast SeqProp generates four CTCF binding sites. For MPRA-DragoNN, we identify both CRE- and CTCF binding sites embedded within a GC-rich context, which aligns well with what we might expect to find in a strong enhancer [57, 58]. Finally, for Optimus 5’, Fast SeqProp generates a T-rich sequence with multiple in-frame (IF) uAUGs. These determinants were found to improve ribosome loading [25]. See the Additional file 1 (Figure S2) for additional visualizations comparing the PWM and Fast SeqProp methods at different stages of optimization.

### Regularized sequence design

**Fig. 3:**
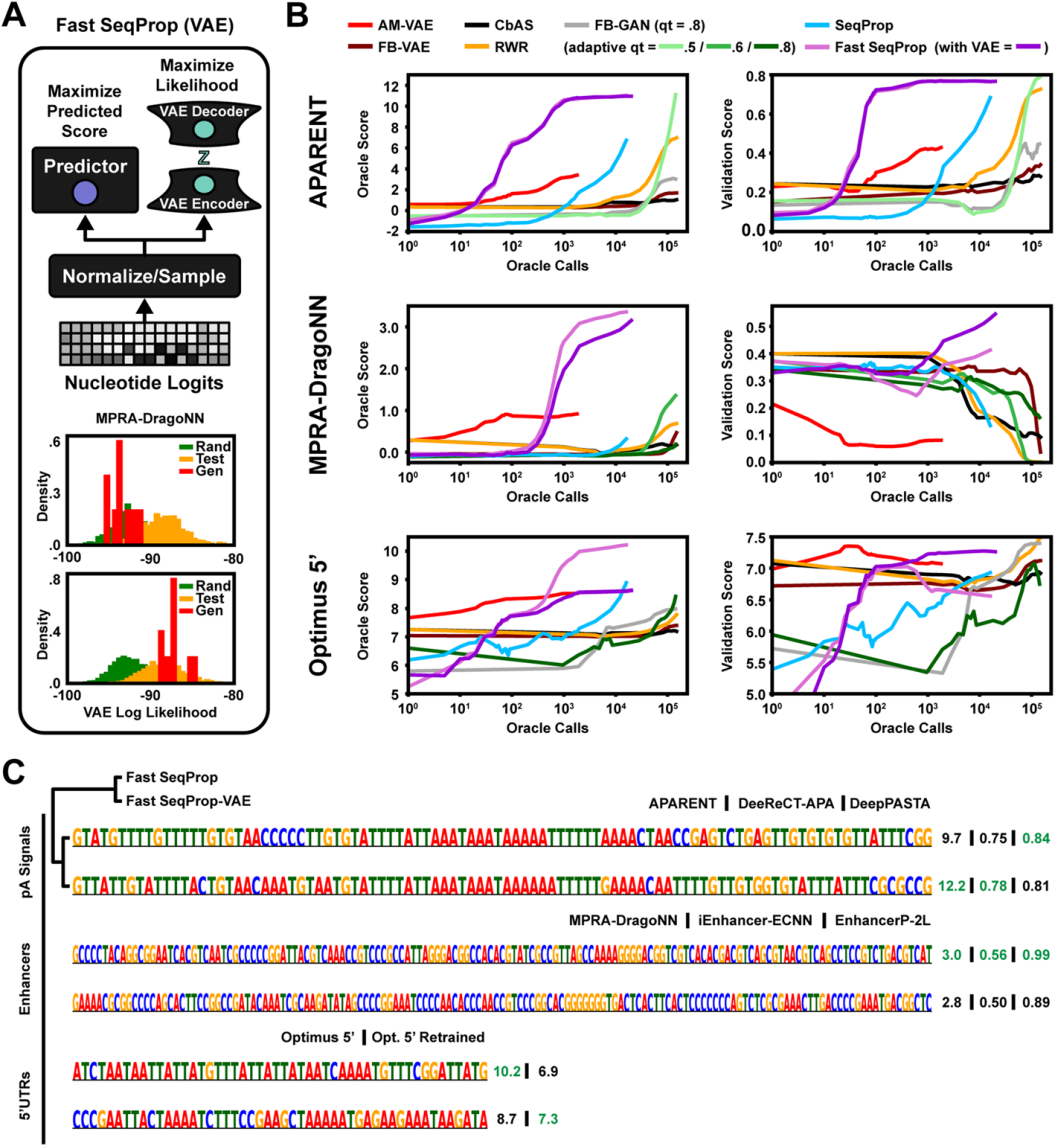
Regularized sequence design. **a** Top: VAE-regularized Fast SeqProp. A variational autoencoder (VAE) is used to control the estimated likelihood of designed sequences during gradient ascent optimization. Bottom: Estimated VAE log likelihood distribution of random sequences (green), test sequences from the MPRA-DragoNN dataset (orange) and designed sequences (red), using Fast SeqProp without and with VAE regularization (top and bottom histogram respectively). **b** Oracle fitness score trajectories (APARENT, MPRA-DragoNN and Optimus 5’) and validation model score trajectories (DeeReCT-APA, iEnhancer-2L and retrained Optimus 5’) as a function of the cumulative number of predictor calls made during the sequence design phase. Shown are the median scores across 10 samples per design method, for three repeats. **c** Example designed sequences for APARENT, MPRA-DragoNN and Optimus 5’, using Fast SeqProp with and without VAE-regularization. Oracle and validation model scores are annotated on the right

While finding regulatory logic in the sequences produced by activation maximization is a good indication that we actually generate patterns with biological meaning, the method may still not be suitable in its most basic form for sequence design. There is the potential issue of overfitting to the predictor oracle during sequence optimization, as the oracle may lose its accuracy when drifting out of the training data distribution to maximize predicted fitness. By training a differentiable likelihood model, such as a variational autoencoder (VAE) [59], on samples from the same data and using it as a regularizer in the cost function, we can prevent drift to low-confidence regions of design space (Fig. 3a; top). Using a VAE to avoid drift has previously been demonstrated by [37, 39, 42]. In summary, we extend the original optimization objective (Eq. 1) by passing the sampled one-hot pattern $\delta (\varvec{l})$ to the VAE and penalize the pattern based on its VAE-estimated marginal likelihood, $p_{\text {VAE}}(\delta (\varvec{l}))$, using importance-weighted inference and ST approximation for backpropagation (see Eq. 13 in Methods).

The degree to which predictors exhibit pathological behavior when maximized varies on a case-by-case basis and likely depends heavily on the data distribution. When designing maximally strong gene enhancers using the MPRA-DragoNN predictor, for example, VAE-regularization has a clear effect on shifting the distribution of the designed sequences (Fig. 3a; bottom histograms). In contrast, when designing polyadenylation signals, VAE-regularization has no effect since non-regularized optimization already generates sequences that are at least as likely as training data according to the VAE (see Additional file 1, Figure S3A).

Next, we tasked the VAE-regularized Fast SeqProp method with designing maximally strong polyadenylation signals (using APARENT as the oracle), maximally transcriptionally active enhancer sequences (using MPRA-DragoNN as the oracle) and maximally translationally efficient 5’ UTRs (using Optimus 5’). For each task, we trained a $\beta$-VAE [59] and a W-GAN [60] on a sample of 5000 high-fitness sequences (see Methods for details). We then used the methods CbAS [39] FB-GAN [38], AM-VAE [44], RWR [61] and FB-VAE (VAE-version of FB-GAN) to maximize each oracle, using the VAE or GAN we trained earlier with default method parameters. We used the same VAE as the regularizer for our design method (Fast SeqProp). During optimization, we measured the fitness scores of both the oracle and a number of independent validation models that we did not directly optimize for, allowing us to estimate sequence fitness in an unbiased way. Specifically, when designing polyadenylation signals based on APARENT, we validated the designs using DeeReCT-APA [31], an LSTM trained on 3’-sequencing data of mouse cells, and DeepPASTA [30], a CNN trained on human 3’-sequencing data. When designing enhancer sequences, we validated the designs using iEnhancer-ECNN [62], an ensemble of CNNs trained on genomic enhancer sequences, and EnhancerP-2L [63], a Random Forest-classifier based on statistical features extracted from enhancer regions in the genome. Finally, to validate Optimus 5’ designs, we had access to a newer version of the model that had been trained on additional MPRA data, making it more robust particularly on outlier sequences such as long homopolymer stretches [25]. On a practical note, we found it difficult to train a VAE on the APARENT, Optimus 5’ and MPRA-DragoNN datasets, and the convergence of CbAS, RWR and FB-GAN appeared sensitive to quantile threshold settings, which we believe stem from the considerable data heterogeneity and variability.

The results (Fig. 3b) show that Fast SeqProp reaches significantly higher oracle fitness scores and validation model scores with orders of magnitudes fewer calls to the oracle for all tasks except the 5’ UTR design problem, where instead AM-VAE reaches high validation scores faster. The other methods either do not reach the same median validation score in the total allotted time, or do so at the expense of reduced diversity (see Additional file 1, Figure S3B). For the polyadenylation signal design task, Fast SeqProp reaches identical validation scores with or without VAE-regularization (Fig. 3b, top right; Additional file 1, Figure S3C). The designed polyadenylation signal sequences include motifs such as CFIm-, CstF- and CPSF binding sites (Fig. 3c, top). For the enhancer design task, the VAE-regularization is clearly beneficial according to the validation model; while enhancers designed by Fast SeqProp without the VAE have a median MPRA-DragoNN score of 3.5, the median iEnhancer-ECNN score (Fig. 3b, middle right) is just 0.43. With VAE-regularization, we generate sequences with a lower median MPRA-DragoNN score (3.25), but higher iEnhancer-ECNN score (0.55). However, closer inspection reveals that Fast SeqProp does not consistently generate worse enhancers according to the validation model than its VAE-regularized counterpart. Rather, Fast SeqProp without VAE either generates highly scored enhancers by the validation model or sequences that are lowly scored, while Fast SeqProp with VAE consistently generates medium-scored enhancers (example shown in Fig. 3c, middle). This dynamic is also observed with another validation model (EnhancerP-2L; see Additional file 1, Figure S3D). Only $80\%$ of Fast SeqProp (no VAE) sequences are identified by EnhancerP-2L as enhancers, while nearly $100\%$ of Fast SeqProp-VAE sequences are identified. However, their weighted predicted enhancer strengths are identical. It is also worth noting that most other methods decrease their validation scores when increasing their MPRA-DragoNN scores; this is because they get stuck in a suboptimal, local minimum with pathological AT-repeats. Finally, VAE-regularization is beneficial for designing 5’ UTRs, as it restricts the sequences from becoming overly T-rich, a sequence pathology present in the original Optimus 5’ model which the retrained version understands actually decreases ribosome load (Fig. 3b, bottom; Fig. 3c, bottom).

In the Additional file 1, we provide extra benchmark experiments comparing Fast SeqProp to a subset of the above design methods. In particular, in Figure S3E, we train the same kind of oracles as was used by Brookes et al. [39] to estimate uncertainty in the fitness predictions [64], and use these models to replicate the polyadenylation signal and 5’ UTR design benchmarks. We also replicate the GFP design task used in Brookes et al. [39]. Additionally, in Figure S3F, we include an example where we use MPRA-DragoNN to design maximally specific enhancers in the cell line HepG2 (and inactivated in K562), and show how internal network penalties can be used to regularize the sequence optimization when it is hard to train an uncertainty-estimator oracle that is sufficiently accurate.

### Protein structure optimization

**Fig. 4:**
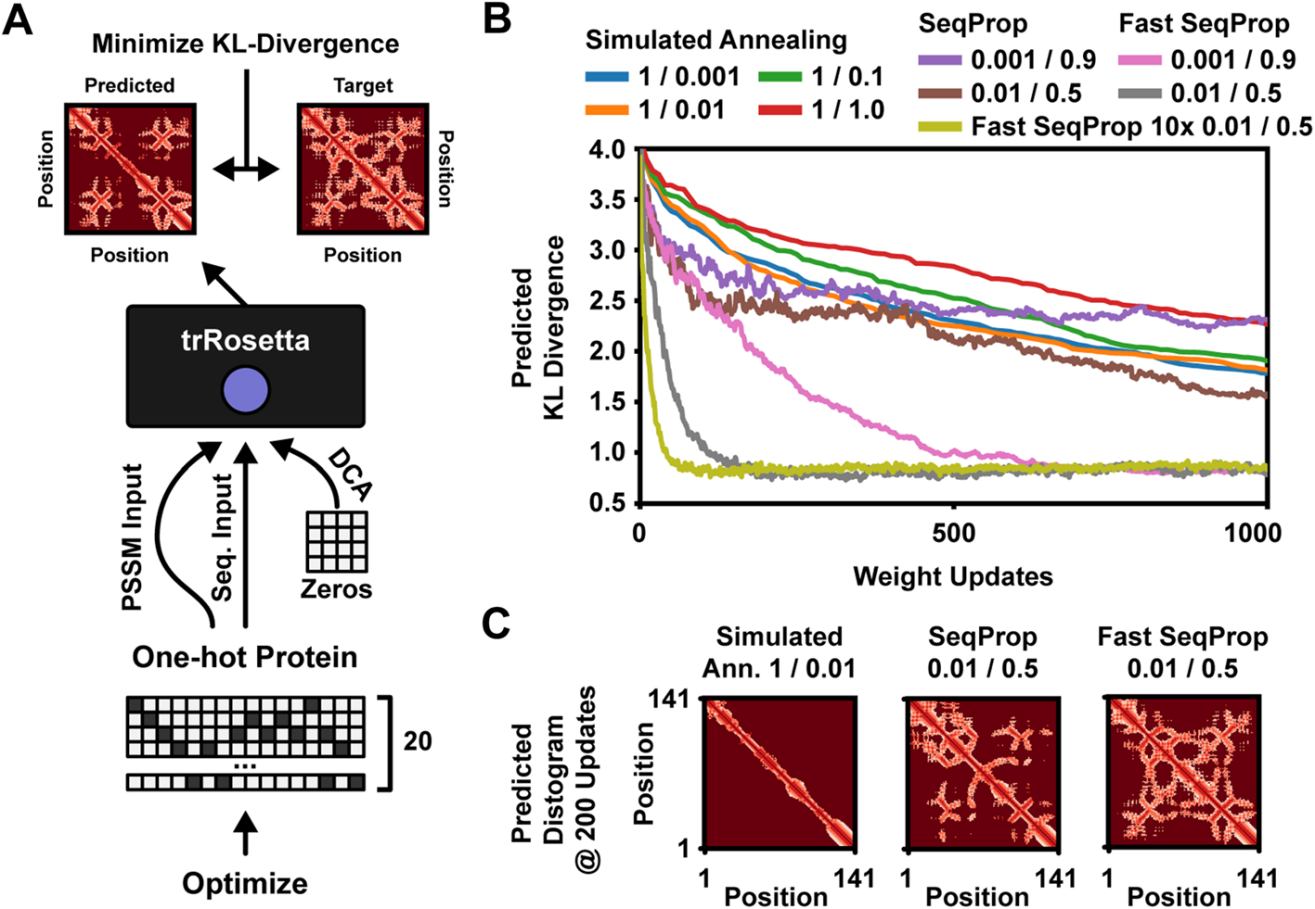
Protein structure optimization. **a** Protein sequences are designed to minimize the KL-divergence between predicted and target distance and angle distributions. The one-hot pattern is used for two of the trRosetta inputs. **b** Generating sequences which conform to the target predicted structure of a Sensor Histidine Kinase. Simulated Annealing was tested at several initial temperatures, with 1 substitution per step. Similarly, SeqProp and Fast SeqProp was tested at several combinations of learning rate and momentum. **c** Predicted residue distance distributions after 200 iterations

Multiple deep learning models have recently been developed for predicting tertiary protein structure [32–34]. Here, we demonstrate our method by designing *de novo* protein sequences which conform to a target residue contact map as predicted by trRosetta [34]. The predictor takes three inputs (Fig. 4a): A one-hot coded sequence, a PSSM constructed from a multiple-sequence alignment (MSA) and a direct-coupling analysis (DCA) map. For our design task, we pass the optimizable one-hot pattern to the first two inputs and an all-zeros tensor as the DCA feature map. Given the predicted distance distribution $\varvec{D}^{P} \in [0, 1]^{N \times N \times 37}$ and angle distributions $\varvec{\theta }^{P}, \varvec{\omega }^{P} \in [0, 1]^{N \times N \times 24}$, $\varvec{\phi }^{P} \in [0, 1]^{N \times N \times 12}$, we minimize the mean KL-divergence against target distributions $\varvec{D}^{T}$, $\varvec{\theta }^{T}$, $\varvec{\omega }^{T}$ and $\varvec{\phi }^{T}$:

9 $$\begin{aligned} \begin{aligned} \min _{\varvec{l}}&\quad \text {KL}(\varvec{D}^{P} || \varvec{D}^{T}) + \text {KL}(\varvec{\theta }^{P} || \varvec{\theta }^{T}) + \text {KL}(\varvec{\omega }^{P} || \varvec{\omega }^{T}) + \text {KL}(\varvec{\phi }^{P} || \varvec{\phi }^{T})\\ \text {where}&\quad \text {KL}(\varvec{X} || \varvec{Y}) = \frac{1}{N^{2}} \cdot \sum _{i=1}^{N} \sum _{j=1}^{N} \sum _{k=1}^{K} \varvec{Y}_{ijk} \cdot \log \bigg ( \frac{\varvec{Y}_{ijk}}{\varvec{X}_{ijk}} \bigg ) \end{aligned} \end{aligned}$$

We compared SeqProp and Simulated Annealing to a modified version of Fast SeqProp, where logits are normalized across all residue channels (*layer*-normalized rather than *instance*-normalized) to reduce the increased variance of shorter sequences with 20 one-hot coded channels. We used the methods to design protein sequences which conformed to the target structure of an example protein (Sensor Histidine Kinase). We optimized 5 independent sequences per design method and recorded the median KL-loss at each iteration. The results show that Fast SeqProp converges considerably faster than other methods (Fig. 4b and Additional file 1 Figure S4A); after 200 iterations, Fast SeqProp reached 4x lower KL-divergence and much of the target structure is visible (Fig. 4c). While the choice of learning rate changes the rate of convergence, it does not alter the minima found by Fast SeqProp. Additionally, by sampling multiple sequences at once and walking down the average gradient (e.g. 10 samples per gradient update), we can improve the rate of convergence further by making the gradient less noisy (see Additional file 1, Figure S4B). Importantly, this scales significantly better than linear in execution time, since multiple samples can be computed and differentiated in parallel on a GPU. Finally, we replicated our results by designing sequences for a different protein structure (an alpha-helical hairpin protein; see Additional file 1, Figure S4C-E).

## Discussion

Methods guided by Machine Learning are used for a growing number of molecular design problems. To support this ongoing effort, it is crucial that we have optimization methods at the sequence-level which are fast, flexible and generally applicable with minimal tuning. *Fast SeqProp* is a model-free method that exhibits many of these properties. We demonstrated the method on a diverse set of problems, including the design of strong polyadenylation signals, efficiently translated 5’ UTRs and enhancers that result in high transcriptional activity. Interestingly, Fast SeqProp found higher fitness optima when compared to estimation-of-distribution (EDA) approaches, in particular for design tasks with low epistemic uncertainty. These results suggest that conditioning of deep generative models might be overly restrictive for some problems.

By normalizing nucleotide logits across positions and using a global entropy parameter, Fast SeqProp keeps logits proportionally scaled and centered at zero. The gradient of the entropy parameter $\gamma$ in our design method adaptively adjusts the sampling temperature to trade off global and local optimization. In the beginning, $\gamma$ is small, corresponding to a high PWM entropy and consequently very diverse sequence samples. As optimization progresses, $\gamma$ grows, leading to more localized sequence changes. This adaptive mechanism, in combination with flexible nucleotide logits due to the normalization, results in a highly efficient design method. As demonstrated on five deep learning predictors, logit normalization enables extremely fast sequence optimization, with a 50-100-fold speedup compared to previous gradient-based methods for many predictors.

In addition to logit drift and vanishing gradients, the original gradient ascent (or activation maximization) method suffers from predictor pathologies due to passing continuous softmax sequence relaxations as input, a problem fully removed by using discrete sampling. We further observed that straight-through sampling leads to consistently better optima than softmax relaxation, suggesting that it traverses local minima. In fact, our method outperformed global optimization meta heuristics such as Simulated Annealing on more difficult design tasks, such as designing 1000 nt long enhancer regions or designing protein sequences which conform to a complex target structure. We further demonstrated robust sequence design even when there is a high degree of epistemic uncertainty, by incorporating a regularization penalty based on variational autoencoders. Our approach showed better and faster convergence than other regularized design methods.

## Conclusion

We presented an improved version of activation maximization for biological sequence design. *Fast SeqProp* combines logit normalization with stochastic nucleotide sampling and straight-through gradients. We demonstrated the efficacy of the method on several DNA, RNA and protein design tasks. We expect this algorithmic improvement to be broadly useful to the research community for biomolecular optimization at the level of primary sequence. The approach introduced here could accelerate the design of functional biomolecules, potentially resulting in novel drug therapies, vaccines, molecular sensors and other bioengineering products.

## Methods

### Activation maximization design methods

In Fig. 1 and throughout the paper, we compare four different activation maximization methods for sequences: (1) *Fast SeqProp* (Our method)—The modified activation maximization method which combines the logit normalization scheme of Eqs. 7–8 with the softmax straight-through estimator of Eqs. 5–6, (2) *PWM*—The original method with continuous softmax-relaxed inputs [44], (3) *SeqProp*—The categorical sampling method described in [29] using the (non-normalized) softmax straight-through gradient estimator, and (4) *Fast PWM*—A logit-normalized version of the softmax-relaxed method.

Starting with a randomly initialized logit matrix $\varvec{l}$, for the methods PWM and Fast PWM we optimize $\varvec{l}$ using the softmax relaxation $\sigma (\varvec{l})$ from Eq. 3. For SeqProp and Fast SeqProp, we optimize $\varvec{l}$ using the discrete nucleotide sampler $\delta (\varvec{l})$ from Eq. 5. We define the optimization loss (or the ’train’ loss) as:

$$\begin{aligned} \mathcal {L}_{\text {train}}(\varvec{l}) = - \mathcal {P}(\varvec{x}(\varvec{l})) \end{aligned}$$

For PWM and Fast PWM, $\varvec{x}(\varvec{l}) = \sigma (\varvec{l})$. For SeqProp and Fast SeqProp, $\varvec{x}(\varvec{l}) = \delta (\varvec{l})$.

For Fast SeqProp we use the scaled, normalized logits $\varvec{l}^{(\text {scaled})}$ (Eqs. 7–8) as parameters for the sampler $\delta$ defined in Eq. 5. As such, we minimize the above loss with respect to $\varvec{l}_{ij}$, $\gamma _{j}$ and $\beta _{j}$ (or $\gamma$ and $\beta$ for proteins). Using the softmax ST estimator from Eq. 6, we arrive at the following gradients for Fast SeqProp:

10 $$\begin{aligned} \frac{\partial \mathcal {P}(\delta (\varvec{l}^{(\text {scaled})}))}{\partial \varvec{l}_{ij}}&= \sum _{k=1}^{M} \frac{\partial \mathcal {P}(\delta (\varvec{l}^{(\text {scaled})}))}{\partial \delta (\varvec{l}^{(\text {scaled})})_{ik}} \cdot \frac{\partial \sigma (\varvec{l}^{(\text {scaled})})_{ik}}{\partial \varvec{l}_{ij}^{(\text {scaled})}} \cdot \gamma _{j} \cdot \frac{\partial \varvec{l}_{ij}^{(\text {norm})}}{\partial \varvec{l}_{ij}} \end{aligned}$$

11 $$\begin{aligned} \frac{\partial \mathcal {P}(\delta (\varvec{l}^{(\text {scaled})}))}{\partial \gamma _{j}}&= \sum _{i=1}^{N} \sum _{k=1}^{M} \frac{\partial \mathcal {P}(\delta (\varvec{l}^{(\text {scaled})}))}{\partial \delta (\varvec{l}^{(\text {scaled})})_{ik}} \cdot \frac{\partial \sigma (\varvec{l}^{(\text {scaled})})_{ik}}{\partial \varvec{l}_{ij}^{(\text {scaled})}} \cdot \varvec{l}_{ij}^{(\text {norm})} \end{aligned}$$

12 $$\begin{aligned} \frac{\partial \mathcal {P}(\delta (\varvec{l}^{(\text {scaled})}))}{\partial \beta {j}}&= \sum _{i=1}^{N} \sum _{k=1}^{M} \frac{\partial \mathcal {P}(\delta (\varvec{l}^{(\text {scaled})}))}{\partial \delta (\varvec{l}^{(\text {scaled})})_{ik}} \cdot \frac{\partial \sigma (\varvec{l}^{(\text {scaled})})_{ik}}{\partial \varvec{l}_{ij}^{(\text {scaled})}} \end{aligned}$$

The gradient equations are very similar for Fast PWM (the logit-normalized PWM method); the only difference is that the discrete sampler $\delta$ in the forward pass is replaced by the standard softmax $\sigma$. Similar design methods were published in parallel with (or shortly after) this work, including an editing method based on the Gumbel-Softmax distribution [45] and other algorithms based on discretized activation maximization [46, 65]. See Figure S1E in the Additional file 1 for a comparison to optimization based on Gumbel-Softmax.

The actual loss (or the ’test’ loss) is evaluated on the basis of discrete sequence samples drawn from the optimized softmax representation $\sigma (\varvec{l})$, regardless of design method. In all four methods, we can use the categorical nucleotide sampler $\delta (\varvec{l})$ to draw sequence samples and compute the mean test loss as:

$$\begin{aligned} \mathcal {L}_{\text {test}}(\{\varvec{l}^{(k)}\}_{k=1}^{K}) = - \frac{1}{K} \frac{1}{S} \sum _{k=1}^{K} \sum _{s=1}^{S} \mathcal {P}(\delta (\varvec{l}^{(k)})^{(s)}) \end{aligned}$$

Here *S* refers to the number of samples drawn from each softmax sequence $\sigma (\varvec{l}^{(k)})$ at every weight update *t*, and *K* is the number of independent optimization runs. In all experiments, we set $K = 10$ and $S = 10$.

In addition to gradient-based methods, we compare Fast SeqProp to discrete search algorithms. The first method is a pairwise nucleotide-swapping search (*Evolution*) [25], where sequence $\varvec{x}$ is mutated with either 1 or, with a 50% chance, 2 random substitutions at each iteration, resulting in a new candidate sequence $\varvec{x}'$. $\varvec{x}'$ is only accepted if $\mathcal {P}(\varvec{x}') > \mathcal {P}(\varvec{x})$. We also tested a well-known meta heuristic—*Simulated Annealing* [66]—which has recently been demonstrated for sequence-level protein design [3]. In Simulated Annealing, mutations are accepted even if they result in lower fitness with probability $P(\varvec{x}', \varvec{x}, T)$, where *T* is a temperature parameter. Here we use the Metropolis acceptance criterion [67]:

$$\begin{aligned} P(\varvec{x}', \varvec{x}, T) = e^{-(\mathcal {P}(\varvec{x}) - \mathcal {P}(\varvec{x}')) / T} \end{aligned}$$

#### Adaptive sampling temperature with fast SeqProp

In Fast SeqProp, the scaling parameter $\gamma _{j}$ adaptively adjusts the sampling entropy to control global versus local optimization. This can be deduced from the gradient components of $\gamma _{j}$ in Eq. 11:

1. $\frac{\partial \mathcal {P}(\delta (\varvec{l}^{(\text {scaled})}))}{\partial \delta (\varvec{l}^{(\text {scaled})})_{ik}}$ is positive for nucleotides which increase fitness and negative otherwise.
2. $\frac{\partial \sigma (\varvec{l}^{(\text {scaled})})_{ik}}{\partial \varvec{l}_{ij}^{(\text {scaled})}}$ is positive when $j=k$ and negative otherwise.
3. $\varvec{l}_{ik}^{(\text {norm})}$ is positive only when we are likely to sample the corresponding nucleotide.

Here, the product of the first two terms, $\frac{\partial \mathcal {P}(\delta (\varvec{l}^{(\text {scaled})}))}{\partial \delta (\varvec{l}^{(\text {scaled})})_{ik}} \cdot \frac{\partial \sigma (\varvec{l}^{(\text {scaled})})_{ik}}{\partial \varvec{l}_{ij}^{(\text {scaled})}}$, is positive if $j = k$ and nucleotide *j* raises fitness or if $j \ne k$ and nucleotide *k* lowers fitness. Put together, the gradient for $\gamma _{j}$ increases when our confidence $\varvec{l}_{ij}^{(\text {norm})}$ in nucleotide *j* is *consistent* with its impact on fitness, such that $\text {sign}\left( \sum _{k=1}^{M} \frac{\partial \mathcal {P}(\delta (\varvec{l}^{(\text {scaled})}))}{\partial \delta (\varvec{l}^{(\text {scaled})})_{ik}} \cdot \frac{\partial \sigma (\varvec{l}^{(\text {scaled})})_{ik}}{\partial \varvec{l}_{ij}^{(\text {scaled})}}\right) = \text {sign}\left( \varvec{l}_{ij}^{(\text {norm})}\right)$. Conversely, *inconsistent* nucleotides decrement the gradient. At the start of optimization, $\gamma _{j}$ is small, leading to high PWM entropy and large jumps in sequence design space. As we sample consistent nucleotides and the entropy gradient $\frac{\partial \mathcal {P}(\delta (\varvec{l}^{(\text {scaled})}))}{\partial \gamma _{j}}$ turns positive, $\gamma _{j}$ increases. Larger $\gamma _{j}$ lowers the entropy and leads to more localized optimization. However, if we sample sufficiently many inconsistent nucleotides, the gradient of $\gamma _{j}$ may turn negative, again raising entropy and promoting global exploration.

Note that, in the context of protein design where we have a single scale $\gamma$ and offset $\beta$, the gradient expressions from Eqs. 11 and 12 are additively pooled across all *M* channels. The argued benefits of instance-normalization above thus holds true for layer-normalization as well.

#### VAE-regularized fast SeqProp

In the main paper (Fig. 3), we use a variational autoencoder (VAE) [59] to regularize the sequence design when running Fast SeqProp. Similar regularization techniques based on VAEs have previously been employed by [37, 39]. The original optimization objective (Eq. 1) is extended by passing the sampled one-hot pattern $\delta (\varvec{l})$ to the VAE and estimating its marginal likelihood, $p_{\text {VAE}}(\delta (\varvec{l}))$, using importance-weighted inference. We then minimize a margin loss with respect to the mean likelihood $p_{\text {ref}}$ of the original training data to keep sequence designs in-distribution, using the Softmax ST estimator to propagate gradients back to $\varvec{l}$:

13 $$\begin{aligned} \min _{\varvec{l}} - \mathcal {P}(\delta (\varvec{l})) + \lambda \cdot \text {max}\big [\log _{10} p_{\text {ref}} - \log _{10} p_{\text {VAE}}(\delta (\varvec{l})) - \rho , 0 \big ] \end{aligned}$$

#### VAE-regularized fast SeqProp with uncertainty-estimation

In the Additional file 1 (Figure S3E), we replicate the benchmark comparison of the main paper (Fig. 3), but we use oracle predictors capable of estimating the uncertainty in their fitness predictions to further regularize the designs [64]. Sequence design based on uncertainty estimators were originally proposed by [39, 68]. Assume that the oracle model predicts the mean $\mu \big [\delta (\varvec{l})\big ]$ and standard deviation $\epsilon \big [\delta (\varvec{l})\big ]$ of fitness scores for the designed (sampled) pattern $\delta (\varvec{l})$. We then use the (differentiable) survival function of the normal distribution to maximize the probability $p_{\mu [\delta (\varvec{l})], \epsilon [\delta (\varvec{l})]}(\mathbbm {Y} > q)$ that the predicted fitness of sequence $\delta (\varvec{l})$ is larger than quantile *q* of the training data:

14 $$\begin{aligned} \min _{\varvec{l}} - \log _{10} p_{\mu [\delta (\varvec{l})], \epsilon [\delta (\varvec{l})]}(\mathbbm {Y} > q) + \lambda \cdot \text {max}\big [\log p_{\text {ref}} - \log _{10} p_{\text {VAE}}(\delta (\varvec{l})) - \rho , 0 \big ] \end{aligned}$$

This fitness objective is known as ’Probability of Improvement’ (PI) [69–71].

#### VAE-regularized fast SeqProp with activity-regularization

In the Additional file 1 (Figure S3F), we use the predictor MPRA-DragoNN to design maximally HepG2-specific enhancer sequences, and use activity-regularization on (some of) the internal layers of the predictor to regularize the optimization. We maximize the predicted fitness score $\mathcal {P}(\delta (\varvec{l}))$ (and minimize the VAE-loss as before) while also minimizing a margin loss applied to the sum of a subset of convolutional activation maps $\mathcal {C}_{k}(\delta (\varvec{l}))$:

15 $$\begin{aligned} \begin{aligned} \min _{\varvec{l}}&- \mathcal {P}(\delta (\varvec{l})) + \lambda \cdot \text {max}\big [\log _{10} p_{\text {ref}} - \log _{10} p_{\text {VAE}}(\delta (\varvec{l})) - \rho , 0 \big ]\\&+ \eta _{1} \cdot \text {max}\big [\mathcal {C}_{1}(\delta (\varvec{l})) - C_{1}, 0 \big ] + ... + \eta _{K} \cdot \text {max}\big [\mathcal {C}_{K}(\delta (\varvec{l})) - C_{K}, 0 \big ] \end{aligned} \end{aligned}$$

### Predictor models

We designed sequences for five distinct DNA- or RNA deep learning predictors. For each of these models, we defined one of their (potentially many) outputs as the classification or regression score $\mathcal {P}(\varvec{x}) \in \mathbb {R}$ to maximize in Eq. 1. We also designed protein sequences according to a 3D protein structure predictor. Here is a brief description of each fitness predictor:

*DragoNN* Predicts the probability of SPI1 transcription factor (TF) binding within a 1000-nt sequence. We define $\mathcal {P}(\varvec{x})$ as the logit score of the network output. The trained model was downloaded from:^1^.

*DeepSEA* [22] Predicts multiple TF binding probabilities and chromatin modifications in a 1000-nt sequence. We define $\mathcal {P}(\varvec{x})$ as the logit score of the CTCF (Dnd41) output. The trained model was downloaded from:^2^.

*APARENT* [29] Predicts proximal alternative polyadenylation isoform abundance in a 206-nt sequence. We define $\mathcal {P}(\varvec{x})$ as the logit score of the network output. The trained model was downloaded from:^3^.

*MPRA-DragoNN* [24] Predicts transcriptional activity of a 145-nt promoter sequence. We define $\mathcal {P}(\varvec{x})$ as the sixth output (SV40) of the ’Deep Factorized’ model. The trained model was downloaded from:^4^.

*Optimus 5’* [25] Predicts mean ribosome load in a 50-nt sequence. $\mathcal {P}(\varvec{x})$ is the (non-scaled) output of the ’evolution’ model. The trained model was downloaded from:^5^.

*trRosetta* [34] Predicts amino acid residue distance distributions and angle distributions of the input primary sequence. We defined the optimization objective as minimizing the mean KL-divergence between the predicted distance- and angle distributions of the designed sequence compared to a target structure (see the definition in Section ’Protein Structure Optimization’ of the main paper). The trained model was downloaded from:^6^.

All optimization experiments were carried out in Keras (Chollet, 2015) using Adam with default parameters [72]. Some predictor models were ported using *pytorch2keras*.

### Validation models

When designing sequences for the predictor models listed in the previous section, we computed validation scores based on the following held-out models (i.e. models we did not explicitly optimize for):

*DeeReCT-APA* [31] Predicts relative isoform abundances for multiple competing polyadenylation signals. The model was trained on mouse 3’ sequencing data. We used the model to score a particular designed polyadenylation signal by predicting its relative use when competing with a strong, fixed distal polyadenylation signal. The model was trained using the code repository at:^7^.

*DeepPASTA* [30] Predicts relative isoform abundance of two competing polyadenylation signals. Several model versions exists, we used the one trained on human brain tissue 3’ sequencing data. To score a particular designed polyadenylation signal, we predicted its relative use when competing with a strong, fixed distal signal. The trained model was downloaded from:^8^.

*iEnhancer-ECNN* [62] Detects genomic enhancer regions and predicts whether it is a weak or strong enhancer. We used the product of these two probability outputs to score each designed enhancer sequence. The model was trained using the code repository at:^9^.

*EnhancerP-2L* [63] Detects genomic enhancer regions and predicts whether it is a weak or strong enhancer. For a sample of generated sequences per design method, we calculated the mean detect/not detect prediction rate, the mean weak/strong prediction rate and the mean p-score. The model was available via a web application at:^10^.

*Retrained Optimus 5’* [25] A retrained version of Optimus 5’, where the training data had been complemented with extreme sequences (such as long single-nucleotide repeats, etc.). The trained model was downloaded from:^11^.

### Auxiliary models

In Fig. 3, we trained a variational autoencoder (VAE) [59] and a generative adversarial network (GAN) [60] on a subset of the data that was originally used to train each of the predictor oracles APARENT, MPRA-DragoNN and Optimus 5’. For each design task, we selected a sample of 5000 sequences with highest observed fitness and a sample of 5000 randomly selected sequences. The VAE, which was based on a residual network architecture [73], was trained on the high-fitness subset of sequences. The W-GAN, which was based on the architecture of Gupta et al. [38], was trained on the random subset of sequences.

### Other design methods

A selection of design methods were used for benchmark comparisons in Fig. 3. Here we describe how they were executed and what parameter settings were used:

*CbAS* [39] The procedure was started from the VAE which had been pre-trained on the high-fitness dataset. It was executed for 150 rounds and, depending on design task, either 100 or 1000 sequences were sampled and used for weighted re-training at the end of each round (whichever resulted in higher fitness scores). The threshold was set to either the 60th or 80th pecentile of fitness scores predicted on the training data (whichever resulted in more stable fitness score trajectories). The VAE was trained for either 1 or 10 epochs at the end of each round (whichever resulted in more stable fitness scores—for some tasks, the fitness scores would drop abruptly after only a few sampling rounds when training the VAE for 10 epochs per round). For the benchmark comparison in the main paper, the standard deviation of the predictions were set to a small constant value ranging between 0.02 and 0.1, depending on application (since none of the pre-trained oracles APARENT, MPRA-DragoNN or Optimus 5’ predicts deviation, we used a small constant deviation that was $\sim 50$x smaller than the maximum possible predicted value). In the Additional file 1, where we use oracles with uncertainty estimation, we also supplied the predicted standard deviation to the CbAS survival function. The code was adapted from:^12^.

*RWR* [61] The procedure was started from the VAE which had been pre-trained on the high-fitness dataset. It was executed for 150 rounds and 100 or 1000 sequence samples were used for weighted re-training at the end of each round (whichever resulted in higher fitness scores). The VAE was trained for 10 epochs each round. The code was adapted from:^13^.

*AM-VAE* [44] This method performs activation maximization by gradient ascent through a pre-trained VAE in order to design sequences. The procedure was started from the VAE which had been pre-trained on the high-fitness dataset. Each sequence was optimized for 2000–5000 updates depending on design task (using the Adam optimizer). A normally distributed noise term was added to the gradients to help overcome potential local minima. The code was adapted from:^14^.

*FB-GAN* [38] The FB-GAN procedure was started from the W-GAN which had been pre-trained on a random sample of sequences. The method was executed for 150 epochs and 960 sequences were sampled and used for feedback at the end of each epoch. We either set the feedback threshold to a fixed value (the 80th percentile of fitness scores predicted on the high-fitness dataset), or we adaptively re-set the threshold to a certain percentile as measured on the 960 sampled sequences at the end of each epoch. The code was adapted from:^15^.

*FB-VAE* [38] A VAE-based version of the FB-GAN. The procedure was started from the VAE which had been pre-trained on the high-fitness dataset. It was executed for 150 epochs and 100 or 1000 sequence samples were used for feedback at the end of each epoch (whichever resulted in higher fitness scores). A fixed threshold was used (either the 60th or 80th percentile as predicted on the high-fitness data). The code was adapted from:^16^.

### Graph tools

All graphs were made with Matplotlib [74].

## Supplementary Information

**Additional file 1.** Supplementary Information, containing additional benchmark comparisons, design results and other analyses.

## Data Availability

All code is available at http://www.github.com/johli/seqprop. External software and data used in this study are listed in the Methods section.

## Abbreviations

5’ UTR: 5’ untranslated region
3’ UTR: 3’ untranslated region
AAV: adeno-associated virus
AM: activation maximization
APA: alternative polyadenylation
CbAS: conditioning by adaptive sampling
CNN: convolutional neural network
DEN: deep exploration network
DCA: direct coupling analysis
DNA: deoxyribonucleic acid
FB-GAN: feedback GAN
GAN: generative adversarial network
KL: Kullback–Leibler
LSTM: long short-term memory
ML: machine learning
MPRA: massively parallel reporter assay
MSA: multiple-sequence alignment
OOD: out-of-distribution
PAS: polyadenylation signal
PSSM: position-specific scoring matrix
PWM: position-weight matrix
RNA: ribonucleic acid
RWR: reward-weighted regression
SeqProp: sequence backpropagation
ST: straight-through
TF: transcription factor
VAE: variational autoencoder
W-GAN: Wasserstein GAN

## Footnotes

1 http://mitra.stanford.edu/kundaje/projects/dragonn/SPI1.classification.model.hdf5.

2 http://deepsea.princeton.edu/media/code/deepsea.v0.94c.tar.gz.

3 https://github.com/johli/aparent/tree/master/saved_models.

4 https://github.com/kundajelab/MPRA-DragoNN/tree/master/kipoi/DeepFactorizedModel.

5 https://github.com/pjsample/human_5utr_modeling/tree/master/modeling/saved_models.

6 https://files.ipd.uw.edu/pub/trRosetta/model2019_07.tar.bz2.

7 https://github.com/lzx325/DeeReCT-APA-repo.

8 https://www.cs.ucr.edu/~aaref001/DeepPASTA_site.html.

9 https://github.com/ngphubinh/enhancers.

10 http://biopred.org/enpred/pred.

11 https://github.com/pjsample/human_5utr_modeling/tree/master/modeling/saved_models.

12 https://github.com/dhbrookes/CbAS/.

13 https://github.com/dhbrookes/CbAS/.

14 https://github.com/dhbrookes/CbAS/.

15 https://github.com/av1659/fbgan.

16 https://github.com/dhbrookes/CbAS/.
