# Supplementary material for: Fast activation maximization for molecular sequence design
Source: BMC Bioinformatics. 2021 Oct 20;22:510. doi: 10.1186/s12859-021-04437-5 (PMC8527647; doi:10.1186/s12859-021-04437-5)
Supplement: Supplementary file 1 — Additional file 1. Supplementary Information, containing additional benchmark comparisons, design results and other analyses. [file 12859_2021_4437_MOESM1_ESM.pdf]

Supplementary Information for Paper: "Fast Activation Maximization for Molecular Sequence Design"

Johannes Linder[1*] and Georg Seelig[1,2]

[1] Paul G. Allen School of Computer Science & Engineering, University of Washington, Seattle, USA
[2] Department of Electrical & Computer Engineering, University of Washington, Seattle, USA
[*] Correspondence: jlinder2@cs.washington.edu

Appendix A: Supplemental Activation Maximization Comparisons

Comparison to Evolutionary Algorithms and Simulated Annealing

We compared Fast SeqProp to the discrete nucleotide-swapping search algorithm 'Evolution' (Sample et al., 2019; see Methods) as well as the global optimization heuristic Simulated Annealing (Figure S1A; Kirkpatrick et al., 1983). We benchmarked the methods on the DragoNN maximization task. The results show that Fast SeqProp significantly outperforms Simulated Annealing (Figure S1B); the best fitness score that Simulated Annealing reached in $20,000$ iterations was reached by Fast SeqProp in less than $1,000$ iterations, marking a 20x speed-up. Interestingly, increasing the number of substitutions at each step of Simulated Annealing initially increases the rate of convergence, but results in worse optima. Furthermore, the pairwise nucleotide-swapping search, Evolution, never makes any improvement on its randomly initialized start sequence, suggesting that DragoNN maximization is a highly non-convex optimization problem which pair-wise nucleotide changes is not powerful enough to solve.

Pathologies of Softmax Relaxation

We hypothesized that some predictors, having been trained only on discrete one-hot coded patterns, may have poor predictive power on continuous-valued softmax sequence relaxations (input to PWM). To test this, we measured both the 'training' loss $\mathcal{L}_{\text{train}}$ (which is based on the softmax sequence input $\sigma(\boldsymbol{l})$ for PWM and discrete samples $\delta(\boldsymbol{l})$ for Fast SeqProp) and test loss $\mathcal{L}_{\text{test}}$ (which is based on discrete samples $\delta(\boldsymbol{l})$ regardless of design method) when maximizing MPRA-DragoNN.

Indeed, maximizing MPRA-DragoNN with PWM leads to a severely overestimated predictor score on the softmax input (Figure S1C; top), as the training loss is more than 6-fold lower than the test loss. Using Fast SeqProp, on the other hand, the training and test losses are identical (Figure S1C; bottom). While the training loss is 2x higher than the training loss of PWM, the test loss is more than 3x lower.

Entropy Penalties and the Gumbel Distribution

Curious whether the gap observed between training and test loss for the PWM method in Figure S1C was caused by high softmax entropy, we tested whether an explicit entropy penalty, $\lambda \cdot \frac{1}{N}\sum_{i=1}^{N}\sum_{j=1}^{M} -\sigma(\boldsymbol{l})_{ij} \cdot \log_2 \sigma(\boldsymbol{l})_{ij}$, would improve the method. We re-optimized sequences for Optimus 5' and DragoNN, such that the mean nucleotide conservation reached at least 1.5/2.0 bits (Figure S1D). Even at low entropy, PWM does not converge to nearly as good minima as Fast SeqProp.

We also compared the performance of our logit-normalized, softmax straight-through design method Fast SeqProp to a version of the method using the Gumbel distribution for sampling (Jang et al., 2016; temperature $\tau = 0.1$; Figure S1E). While the Gumbel variant of the design method reached the same optima as Fast SeqProp, it converged slower. Importantly, same as PWM and SeqProp, the Gumbel design method benefited substantially from logit normalization.

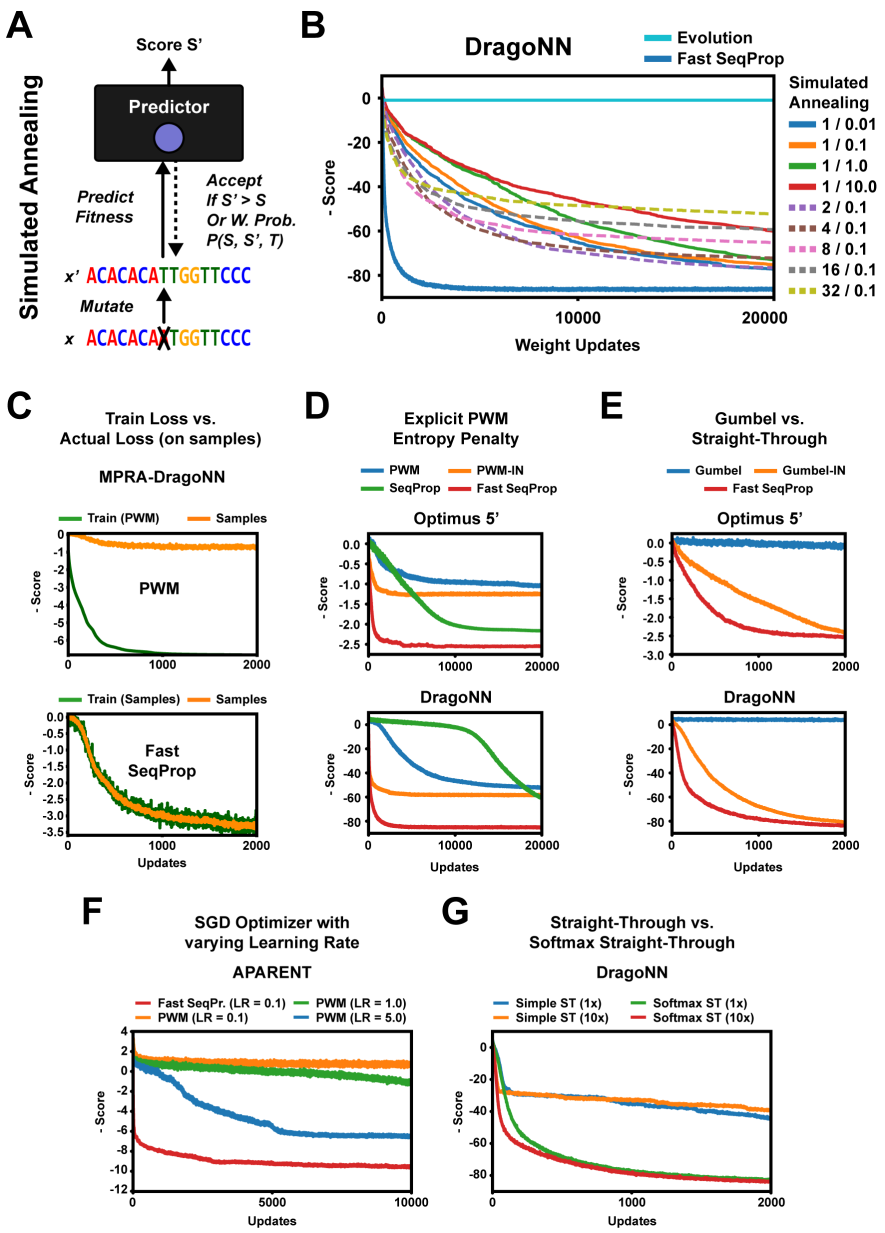

Figure S1 (A) In Simulated Annealing, mutations are accepted with a temperature-controlled probability even if the predicted fitness decreases. (B) Maximizing DragoNN SPI1. Simulated Annealing is tested at several parameter configurations (number of substitutions per step / initial temperature). (C) Maximizing MPRA-DragoNN using either (top) PWM or (bottom) Fast SeqProp as the design method. Shown are the optimization scores during the design phase (green) when we pass either the softmax sequence (PWM) or a sampled sequence (Fast SeqProp) to the predictor, and the corresponding scores if we were to sample sequences from the softmax representation (orange). (D) Maximizing Optimus 5' and DragoNN with a softmax sequence entropy penalty. (E) Comparing Fast SeqProp to a version of the same method using the Gumbel distribution ('Gumbel' – Gumbel-sampling, 'Gumbel-IN' – Gumbel-sampling with instance norm.). (F) Maximizing APARENT using Fast SeqProp or PWM with SGD (LR = Learning Rate). (G) Maximizing DragoNN using Fast SeqProp, with either the softmax- or original (simple) straight-through estimator. 1x and 10x refer to the number of sequences sampled at each update.

Insensitivity to Optimizer Settingss

We noted that the performance of the PWM method was dependent on optimizer settings (Figure S1F); the speed at which it could maximize APARENT was increased by switching to an SGD optimizer and

setting a very high learning rate. However, the method could still not reach the same optimum as Fast SeqProp, which operated well at a default SGD learning rate of 0.1.

Superiority of Softmax Straight-Through Gradients

We compared the performance of Fast SeqProp, which uses the softmax straight-through gradient estimator $\frac{\partial \delta(\boldsymbol{l})_{ij}}{\partial \boldsymbol{l}_{ik}} = \frac{\partial \sigma(\boldsymbol{l})_{ij}}{\partial \boldsymbol{l}_{ik}} = \sigma(\boldsymbol{l})_{ik} \cdot (\mathbb{1}_{(j=k)} - \sigma(\boldsymbol{l})_{ij})$, to a version using the original estimator $\frac{\partial \delta(\boldsymbol{l})_{ij}}{\partial \boldsymbol{l}_{ij}} = 1$. As demonstrated on DragoNN, the softmax estimator reaches much better optima (Figure S1G). Sampling multiple sequences $\{\delta(\boldsymbol{l})^{(s)}\}_{s=1}^{S}$ at each logit update and walking down the average gradient $\frac{1}{S}\sum_{s=1}^{S} \nabla_{\boldsymbol{l}} \mathcal{P}(\delta(\boldsymbol{l})^{(s)})$ slightly speeds up convergence, but does not improve optima.

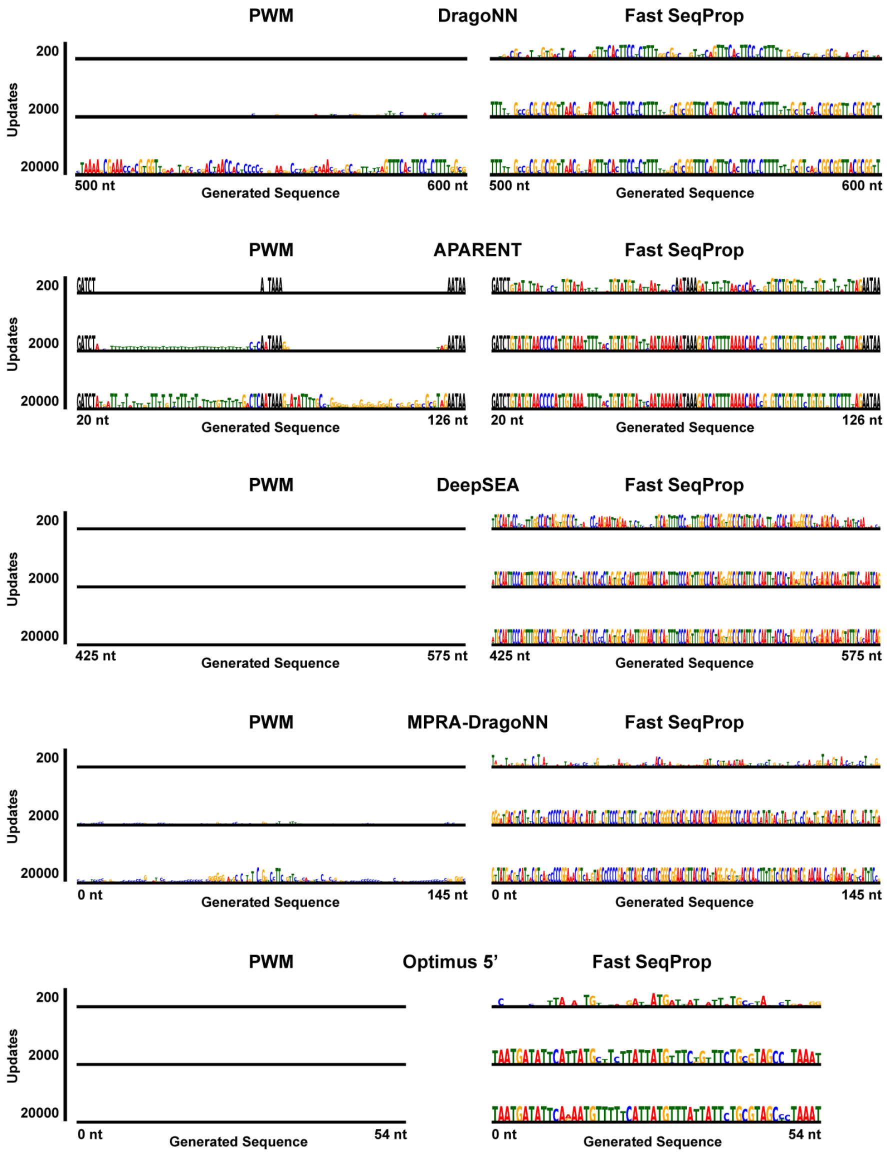

Figure S2 Generation of sequences which maximize the score of DragoNN (SPI1), APARENT, DeepSEA (CTCF, Dnd41), MPRA-DragoNN (SV40 Mean activity) and Optimus 5'. Shown are the resulting sequence logos of running the PWM and Fast SeqProp optimization methods for 200, 2,000 and 20,000 iterations respectively, starting from randomly initialized logits.

Appendix B: Additional Sequence Optimization Examples

Figure S2 depicts an example optimization run for each predictor, comparing the softmax sequences $\sigma(\boldsymbol{l})$ (PSSMs) generated by the PWM and Fast SeqProp design methods. The same sequences as those shown in Figure 2 are also shown here, but for 200, $2,000$ and $20,000$ iterations of gradient ascent (Adam). The logit matrices $\boldsymbol{l}$ were uniformly randomly initialized and default Adam parameters were used.

Appendix C: Additional Sequence Design Comparisons

In Figure S3A-D we report additional performance metrics tracked for each of the benchmarked methods when designing sequences for APARENT (polyadenylation signals), MPRA-DragoNN (transcriptional enhancers) and Optimus 5' (translationally efficient 5' UTRs). We measure median edit distance between designed sequences in Figure S3B as a proxy for diversity. In Figure S3C we measure the strength (relative probability of selection) of designed polyadenylation signals using the held-out model DeepPASTA. In Figure S3D, we measure the strength of designed enhancer sequences using the held-out model EnhancerP-2L.

In Figure S3E we perform a benchmark analysis comparing Fast SeqProp to the methods CbAS (Brookes et al., 2019), AM-VAE (Killoran et al., 2017), FB-VAE (Gupta et al., 2019) and RWR (Peters et al., 2007). The benchmark is very similar to the one presented in the main paper (Figure 3B), but instead of using the pre-trained oracle predictors APARENT, MPRA-DragoNN and Optimus 5' we here train new probabilistic oracles based on the loss function from Lakshminarayanan et al. (2017). These models are capable of estimating the uncertainty in their fitness predictions, a property that can be used to regularize the sequence optimization based on predictor confidence. Specifically, for Fast SeqProp, we use the (differentiable) survival function of the normal distribution in order to maximize the probability that the predicted fitness of designed sequences is larger than a particular quantile q of the training data (see Equation 14 in Methods). The survival function is also used as part of the sample re-weighting scheme in CbAS (Brookes et al., 2019). In our experiments, we use $q = 0.95$.

We carried out the benchmark on three design tasks: (1) Polyadenylation (pA) signal design (by training the oracle on the same data as APARENT was trained on), (2) 5' UTR design (by training the oracle on the same data as Optimus 5') and (3) Green Fluorescent Protein (GFP) variant design (replicating the test suite from Brookes et al., 2019). For pA signal design and 5' UTR design, we used a single oracle model for each design task, consisting of two convolutional layers and a single fully connected hidden layer. After training, the oracle predictions were highly correlated with measured fitness on held-out data (pearson $r = 0.87$ and 0.91 for each respective task). For the GFP design task, we used an ensemble of 5 predictors (each consisting of a single fully connected hidden layer). We used DeeReCT-APA to validate the pA design task (Li et al., 2020). We used the retrained version of Optimus 5' to validate the 5' UTRs (Sample et al., 2019). Finally, we used the same GP regression model as was used by Brookes et al. (2019) to validate the GFP task. When running CbAS and FB-VAE, we used $q = 0.8$ as the quantile cutoff. For CbAS, FB-VAE and RWR, we used 1000 samples per re-weighting/feedback round except for the GFP task where 100 samples were used. The same VAE models that were used in the main paper were also used here for pA signal and 5' UTR design.

As shown in Figure S3E, for the pA signal task, Fast SeqProp designs sequences with higher validation scores than all other methods. Both Fast SeqProp and AM-VAE converge to higher scores than CbAS, FB-VAE and RWR using 100x-$1,000$x fewer calls to the oracle. For the 5' UTR design task, RWR ultimately reaches marginally higher validation score than Fast SeqProp, but does so after 100x more oracle calls. Fast SeqProp reaches higher validation scores than all other methods. Interestingly, for AM-VAE (which is also based on activation maximization), the validation scores decrease throughout the course of optimization. Finally, for the GFP task, methods AM-VAE, CbAS, FB-VAE and RWR

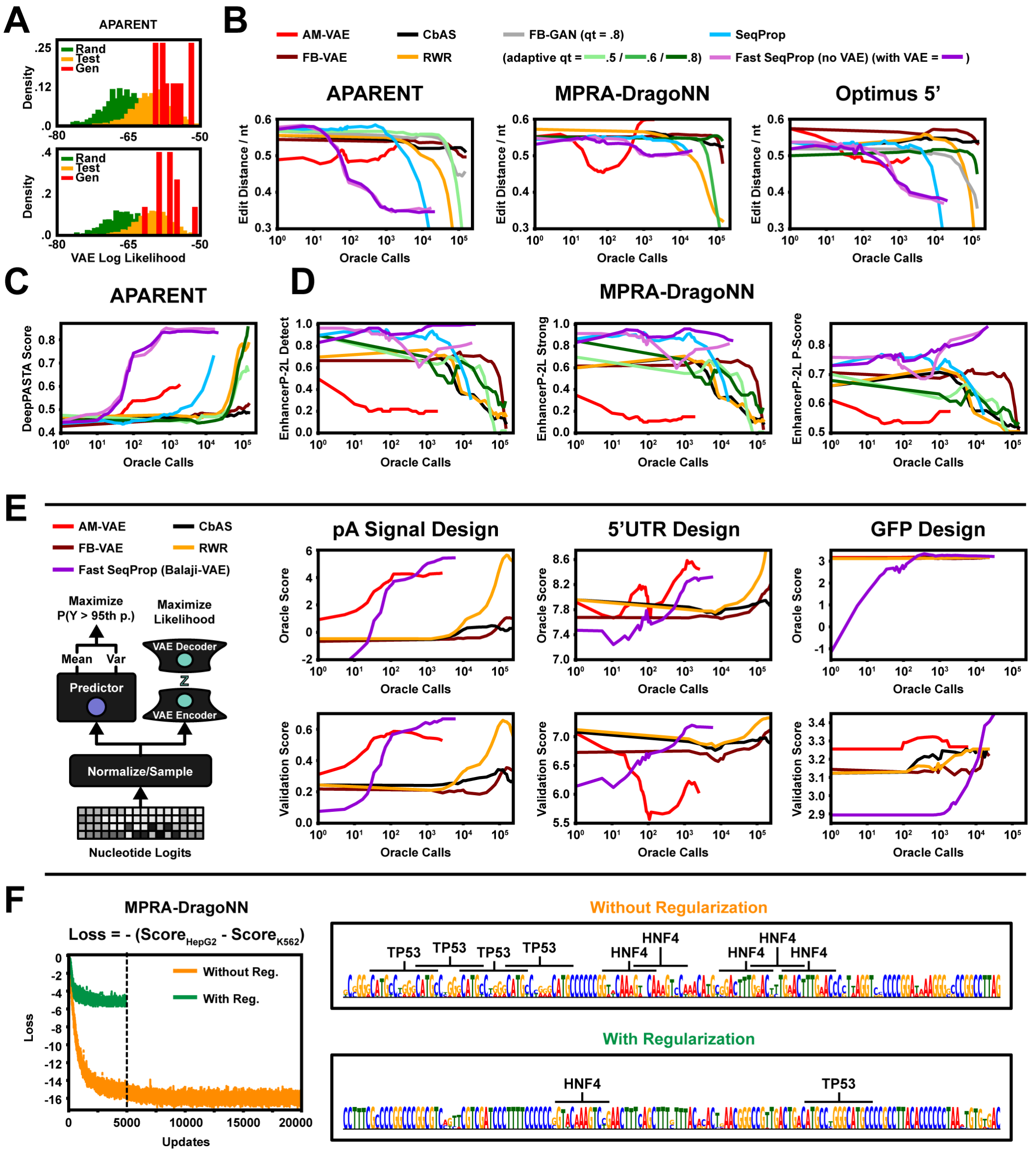

Figure S3 (A) Estimated VAE log likelihood distribution of random sequences (green), test sequences from the APARENT dataset (orange) and designed sequences (red), using Fast SeqProp without and with VAE regularization (top and bottom histogram respectively). (B) The generated sequence diversity of each design methods as a function of the cumulative number of predictor calls, where diversity is measured as the median relative edit distance between each of the 10 optimized sequence samples per method, across three repeats. (C) Median predicted relative use of the designed polyadenylation signals for each method, using the DeepPASTA model. Median score reported across three repeats. (D) Three predicted types of validation scores using the EnhancerP-2L model: (left) The mean frequency of identified functional enhancers, (middle) the mean weighted frequency of identified enhancers (weak enhancers are worth 0.5, strong enhancer 1.0), and (right) Predicted EnhancerP-2L P-scores. The means are computed across three repeats. (E) Additional benchmark analysis using both VAE-regularization and a probabilistic oracle ensemble capable of estimating uncertainty to design sequences (Lakshminarayanan et al., 2017; see Appendix C for details). Top: Median oracle fitness scores across three repeats for the tasks: pA signal design, 5' UTR design and GFP variant design (Brookes et al., 2019); Bottom: Median validation scores across three repeats, using DeeReCT-APA, Optimus 5' (retrained) and a GP regression model respectively. (F) Maximizing the difference in transcriptional activity between cell lines K562 and HepG2 (predicted by MPRA-DragoNN). Shown are the loss and an example designed sequence when running Fast SeqProp without any regularization (orange) and when applying both VAE- and Activity-regularization (green; see Appendix C for details). Discovered motifs annotated above each example sequence.

initially start with significantly higher oracle- and validation scores than Fast SeqProp. The reason is that these methods start by sampling sequences from the pre-trained VAE, which already has high validation scores. Throughout the course of optimization, these methods marginally increase their validation scores. However, after $\sim 1,000$ predictor calls, Fast SeqProp quickly converges to sequence designs with higher validation scores than all other methods.

We tried using the above approach on the MPRA-DragoNN enhancer data from Ernst et al., (2016), but we failed to train a sufficiently accurate oracle predictor with the loss function from Lakshminarayanan et al. (2017). This is likely due to the substantial levels of noise present in the training data (as is also described by Movva et al., 2019). This enhancer dataset is unique in that it contains replicate functional measurements in multiple cell types (K562 and HepG2) and in theory would allow the design of cell-type specific enhancer sequences. However, if we cannot even fit the oracle model to individual cell type measurements, we have no hope of fitting the oracle to differences between two (noisy) cell type readouts (which would enable cell-type specific design). When we attempted this, we ended up with a very weak oracle (pearson $r = 0.05$ on held-out data, using the difference in measured transcriptional activity between HepG2 and K562 with SV40 promoters as target value).

Instead, we used the original MPRA-DragoNN predictor, but with regularization penalties applied to the sum of activations across some of the model's internal convolutional layers in order to design robust, HepG2-specific enhancers (see Equation 15 in Methods). Specifically, we maximized the predicted cell-type specificity $\mathcal{P}(\boldsymbol{x})^{(\text{HepG2})} - \mathcal{P}(\boldsymbol{x})^{(\text{K562})}$ while also minimizing a margin loss applied to the sum of the 5:th, 8:th and 9:th convolutional activation maps (and also minimizing the VAE-loss as before). Figure S3F shows the result of using Fast SeqProp to design maximally HepG2-specific enhancers, with and without regularization. Without regularization, the design method fills an example designed sequence with 4 TP53 binding motifs and 5 HNF4 binding motifs. With regularization, only one copy of each motif appears in the designed example.

Appendix D: Extra Protein Structure Optimization Example

The categorical KL-divergence measured in Figure 4B of the main paper is not necessarily a good metric for estimating the distance between an optimized protein structure and its target structure. For example, if the optimized structure puts all of its probability mass just one discretized bin away in the predicted distance map $\boldsymbol{D}^P_{ijk+1}$ compared to the target distance map $\boldsymbol{D}^T_{ijk}$, the KL-divergence becomes nearly maximal even though the actual distances between structures is quite small.

To mitigate this issue, we validate the optimized structures using a 'smooth' KL-divergence metric, which transforms the discretized, binned distance maps $\boldsymbol{D}$ and angle distributions $\boldsymbol{\theta}, \boldsymbol{\omega}, \boldsymbol{\phi}$ into a single weighted probability at each position i, j. For $\boldsymbol{\theta}$ and $\boldsymbol{\omega}$, we use sin- and cos transforms in order to take into account that bins $\boldsymbol{\theta}_{ij1}$ and $\boldsymbol{\theta}_{ijK}$ are close in the unit circle. We also take into account that, in the distance and angle distributions predicted by trRosetta, bin 0 has the special meaning 'no contact'. The smooth KL-divergence $\mathcal{L}_{\text{smooth}}\big(\boldsymbol{D}^P, \boldsymbol{\theta}^P, \boldsymbol{\omega}^P, \boldsymbol{\phi}^P\big)$ is computed according to the following formulas:

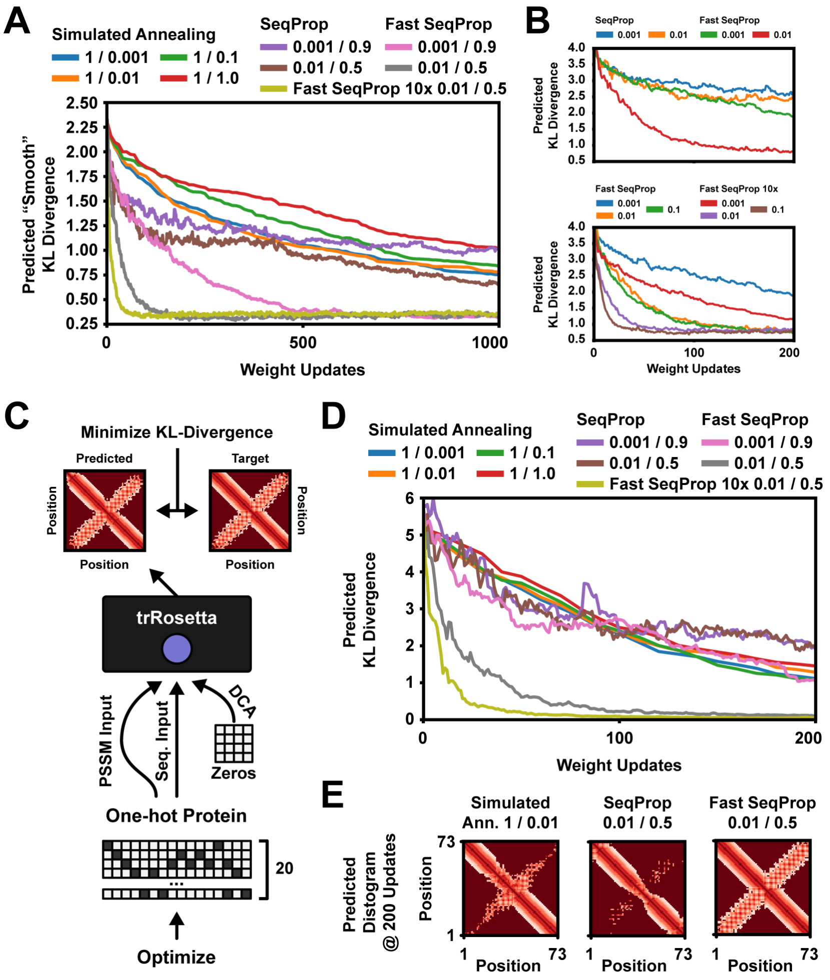

Figure S4 (A) Identical optimization as the one shown in Figure 4B, but here we report the "Smooth" KL divergence, as described in Appendix D. (B) The effect of various Adam learning rate- and momentum parameter settings during structure optimization. "10x" refers to sampling 10 sequences at each step of Fast SeqProp and walking down the average gradient. (C) Sequences are designed to conform to a coiled-coil hairpin structure, as predicted by trRosetta. (D) Sequence optimization results after 1000 iterations. Simulated Annealing was tested at several initial temperatures and SeqProp / Fast SeqProp was tested with multiple optimizer parameters (Adam). (E) Predicted residue distance distributions after 200 iterations.

$$\mathcal{L}_{\text{smooth}}\left(\boldsymbol{D}^P, \boldsymbol{\theta}^P, \boldsymbol{\omega}^P, \boldsymbol{\phi}^P\right) = \text{KL}_{\text{smooth}}(\boldsymbol{D}^P||\boldsymbol{D}^T) + \text{KL}_{\text{smooth}}(\boldsymbol{\phi}^P||\boldsymbol{\phi}^T) + \text{KL}_{\text{circular}}(\boldsymbol{\theta}^P||\boldsymbol{\theta}^T) + \text{KL}_{\text{circular}}(\boldsymbol{\omega}^P||\boldsymbol{\omega}^T)$$

$$\text{KL}_{\text{smooth}}(\boldsymbol{X}||\boldsymbol{Y}) = \frac{1}{N^2} \cdot \sum_{i=1}^{N}\sum_{j=1}^{N} \left(\text{Smooth}(\boldsymbol{Y})_{ij} \cdot \log\left(\frac{\text{Smooth}(\boldsymbol{Y})_{ij}}{\text{Smooth}(\boldsymbol{X})_{ij}}\right) + \boldsymbol{Y}_{ij0} \cdot \log\left(\frac{\boldsymbol{Y}_{ij0}}{\boldsymbol{X}_{ij0}}\right) + \boldsymbol{\epsilon}(\boldsymbol{Y})_{ij} \cdot \log\left(\frac{\boldsymbol{\epsilon}(\boldsymbol{Y})_{ij}}{\boldsymbol{\epsilon}(\boldsymbol{X})_{ij}}\right)\right)$$

$$\text{KL}_{\text{circular}}(\boldsymbol{X}||\boldsymbol{Y}) = \frac{1}{N^2} \cdot \sum_{i=1}^{N}\sum_{j=1}^{N} \left(\text{Circular}(\boldsymbol{Y})_{ij} \cdot \log\left(\frac{\text{Circular}(\boldsymbol{Y})_{ij}}{\text{Circular}(\boldsymbol{X})_{ij}}\right) + \boldsymbol{Y}_{ij0} \cdot \log\left(\frac{\boldsymbol{Y}_{ij0}}{\boldsymbol{X}_{ij0}}\right) + \boldsymbol{\eta}(\boldsymbol{Y})_{ij} \cdot \log\left(\frac{\boldsymbol{\eta}(\boldsymbol{Y})_{ij}}{\boldsymbol{\eta}(\boldsymbol{X})_{ij}}\right)\right)$$

$$
\begin{aligned}
\mathrm{Smooth}(\boldsymbol{X})_{ij} &= \sum_{k=1}^{K}\left(\frac{k-1}{K-1}\right)\cdot \boldsymbol{X}_{ijk} \\
\mathrm{Circular}(\boldsymbol{X})_{ij} &= \sum_{k=1}^{K} 0.5\cdot\left[\left(\sin\left[\left(\frac{k-1}{K-1}\right)\cdot 2\pi-\pi\right]\cdot 0.5+0.5\right)\cdot \boldsymbol{X}_{ijk}\right] \\
&\quad + \sum_{k=1}^{K} 0.5\cdot\left[\left(\cos\left[\left(\frac{k-1}{K-1}\right)\cdot 2\pi-\pi\right]\cdot 0.5+0.5\right)\cdot \boldsymbol{X}_{ijk}\right] \\
\boldsymbol{\epsilon}(\boldsymbol{X})_{ij} &= 1-\mathrm{Smooth}(\boldsymbol{X})_{ij}-\boldsymbol{X}_{ij0} \\
\boldsymbol{\eta}(\boldsymbol{X})_{ij} &= 1-\mathrm{Circular}(\boldsymbol{X})_{ij}-\boldsymbol{X}_{ij0}
\end{aligned}
$$

In addition to the protein structure design task evaluated in Figure 4, we also benchmarked SeqProp, Fast SeqProp and Simulated Annealing on a separate protein structure. Here, we task the methods with designing sequences conforming to a coiled-coil hairpin structure, again using trRosetta as the differentiable structure predictor (Yang et al., 2020). The same KL-divergence loss as was used in Figure 4 was used here. The results, depicted in Figure S4C-E, show that Fast SeqProp converges very quickly to a near-optimal (zero) KL-divergence. The structure is likely easier to design sequences for, as there is only one major long-ranging contact formation. The sequence is also only about half as long as the one in Figure 4.

References

Brookes, D.H., Park, H. and Listgarten, J., 2019. Conditioning by adaptive sampling for robust design (arXiv).

Ernst, J., Melnikov, A., Zhang, X., Wang, L., Rogov, P., Mikkelsen, T.S. and Kellis, M., 2016. Genome-scale high-resolution mapping of activating and repressive nucleotides in regulatory regions. Nature biotechnology 34, 1180-1190.

Gupta, A. and Zou, J., 2019. Feedback GAN for DNA optimizes protein functions. Nature Machine Intelligence 1, 105-111.

Jang, E., Gu, S. and Poole, B., 2016. Categorical reparameterization with gumbel-softmax (arXiv).

Killoran, N., Lee, L. J., Delong, A., Duvenaud, D. and Frey, B. J., 2017. Generating and designing DNA with deep generative models (arXiv).

Kirkpatrick, S., Gelatt, C. D. and Vecchi, M. P., 1983. Optimization by simulated annealing. Science 220, 671-680.

Lakshminarayanan, B., Pritzel, A. and Blundell, C., 2017. Simple and scalable predictive uncertainty estimation using deep ensembles. Advances in neural information processing systems, 30, 6402-6413.

Li, Z., Li, Y., Zhang, B., Li, Y., Long, Y., Zhou, J., Zou, X., Zhang, M., Hu, Y., Chen, W. and Gao, X., 2021. Deerect-apa: Prediction of alternative polyadenylation site usage through deep learning. Genomics, Proteomics & Bioinformatics.

Movva, R., Greenside, P., Marinov, G. K., Nair, S., Shrikumar, A. and Kundaje, A., 2019. Deciphering regulatory DNA sequences and noncoding genetic variants using neural network models of massively parallel reporter assays. PloS One 14.

Peters, J. and Schaal, S., 2007, June. Reinforcement learning by reward-weighted regression for operational space control. In Proceedings of the 24th international conference on Machine learning, 745-750.

Sample, P. J., Wang, B., Reid, D. W., Presnyak, V., McFadyen, I. J., Morris, D. R. and Seelig, G., 2019. Human 5' UTR design and variant effect prediction from a massively parallel translation assay. Nature Biotechnology 37, 803-809.

Yang, J., Anishchenko, I., Park, H., Peng, Z., Ovchinnikov, S. and Baker, D., 2020. Improved protein structure prediction using predicted interresidue orientations. Proceedings of the National Academy of Sciences.
